# Unilateral versus bilateral percutaneous kyphoplasty for osteoporotic vertebral compression fractures: A meta-analysis

**DOI:** 10.1097/MD.0000000000006738

**Published:** 2017-04-28

**Authors:** Wenli Chang, Xinyan Zhang, Ning Jiao, Peizhi Yuwen, Yanbin Zhu, Fei Zhang, Wei Chen

**Affiliations:** a Department of Orthopaedic Surgery; b Department of Radiology; c Department of Pharmacy, the Third Hospital of Hebei Medical University, Shijiazhuang, P.R. China.

**Keywords:** bilateral percutaneous kyphoplasty, meta-analysis, osteoporotic vertebral compression fractures, unilateral percutaneous kyphoplasty

## Abstract

**Background:**

The debate on the efficacy of unilateral percutaneous kyphoplasty (UPKP) and bilateral percutaneous kyphoplasty (BPKP) for the treatment of osteoporotic vertebral compression fractures (OVCFs) is ongoing.

This meta-analysis aimed to evaluate the clinical results of UPKP and BPKP in the treatment of OVCFs.

**Methods:**

Web of Science, PubMed, Embase, and the Chinese Biomedical Database publication databases were searched using a date range of January 2008 to November 2016, for studies comparing UPKP and BPKP for the treatment of OVCFs. The clinical effectiveness was assessed by comparing perioperative outcomes (surgery time, the volume of injected cement, X-ray exposure time, and kyphotic angle reduction), clinical outcomes (visual analogue scale [VAS] for pain relief and Oswestry Disability Index [ODI] for quality of life), and surgery-related complications (cement leakage and adjacent vertebral fractures). Data were analyzed using Stata/SE11.0 software.

**Results:**

Fourteen trials with 1194 patients were retrieved. The pooled results showed significant differences in surgery time (weighted mean difference [WMD] −21.44, 95% confidence interval [CI] [−23.57 to −19.30]; *P* < .001); volume of injected cement [WMD −1.90, 95% CI [−2.26 to −1.54); *P* < .001); and X-ray exposure time (WMD −13.66, 95%CI [−19.59 to −7.72]; *P* < .001) between UPKP and BPKP treatments. However, the pooled results showed no significant differences in kyphotic angle reduction, VAS in the short-term, VAS in the long-term, ODI, cement leakage, or adjacent vertebral fractures between the 2 surgical procedures. Following a subgroup analysis, the results based on randomized controlled trials (RCTs) indicated that there were significant differences in surgery time (WMD −24.65, 95%CI [−26.53 to −22.77]; *P* < .001) and the volume of injected cement (WMD −1.66, 95%CI [−1.97 to −1.36]; *P* < .001) between UPKP and BPKP treatment procedures, respectively. The results based on RCTs indicated that there were no significant differences, either in kyphotic angle reduction or in X-ray exposure time, between the 2 surgical procedures.

**Conclusions:**

Compared to BPKP procedures, UPKP procedures may achieve similar clinical results in the treatment of OVCFs when assessed in terms of the pain relief, improvements in life quality, and surgery-related complications. However, UPKP procedures had a shorter operation time and volume of injected cement compared with BPKP procedures. Additional high quality and multicenter RCTs are needed to provide further robust evidence.

## 1 Introduction

Vertebral compression fractures are the most common fractures that occur in patients with osteoporosis, affecting approximately 20% of individuals older than 70 years.^[1]^ Osteoporotic vertebral compression fractures (OVCFs), especially those that are not treated properly, can lead to deformity, disability, and poor quality of life. Traditionally, patients with OVCFs have been treated with bed rest, physical therapy, analgesics, and the use of an external brace, options that all often have limited effectiveness, especially in elderly patients who have an increased risk of pneumonia, decubitus ulcers, and venous thromboembolism.^[2]^ Furthermore, some patients who do not respond to these conservative treatments turn to surgical treatment, which is typically reserved for OVCFs coupled with neurological deficits or spinal instabilities.^[3]^ Vertebroplasty and balloon kyphoplasty are 2 treatments that were developed for the management of symptomatic OVCFs. Both percutaneous vertebroplasty and balloon kyphoplasty can increase bone strength as well as alleviate the pain caused by OVCFs. However, Buchbinder et al performed 2 multicenter, randomized, double-blind, placebo-controlled trials and found no beneficial effect of vertebroplasty compared with a sham procedure in patients with painful osteoporotic vertebral fractures at 1 week or at 1, 3, 6, 12, or 24 months after treatment.^[4,5]^ Because meta-analysis can offer the highest level of scientific evidence about the efficacy of an intervention based on the principles of evidence-based medicine, in 2015, Buchbinder et al included 11 randomized controlled trials (RCTs) and 1 quasi-RCT conducted in various countries in another study.^[6–8]^ This review did not support a role for vertebroplasty in the treatment of osteoporotic vertebral fractures in routine practice. In addition, this procedure was indicated to be incapable of restoring the initial height of the vertebral body.^[9]^

Conversely, height deformity can be corrected by percutaneous balloon kyphoplasty. Specifically, percutaneous balloon kyphoplasty was initially introduced for tumoral and osteoporotic lesions, and the procedure was later adapted for the management of vertebral fractures.^[10]^ In balloon kyphoplasty, an inflatable bone tamp is used to raise the vertebral body height followed by injection of polymethyl methacrylate for mechanical stabilization.^[11]^ Wang et al^[12]^ determined that kyphoplasty was a safe and effective surgical procedure in treating OVCFs and offered a less injected cement volume, better short-term pain relief, better improvement of short- and long-term kyphotic angle, and a lower cement leakage rate. This technique consists of unilateral percutaneous kyphoplasty (UPKP) and bilateral percutaneous kyphoplasty (BPKP) procedures.

In recent years, some meta-analyses have been conducted to evaluate the advantages and disadvantages of the use of UPKP and BPKP in treating OVCFs.^[13–16]^ However, although the 2 latest meta-analyses have updated results from the previous reviews, they did not investigate the potential heterogeneity in the sources through either sensitivity analysis or subgroup analysis, which may weaken the reliability of the pooled results, especially if there was large heterogeneity.^[13,14]^ There have been additional studies since those reviews were conducted that may further enrich the final results, however. Therefore, this study is intended to evaluate the clinical results of UPKP and BPKP for the treatment of OVCFs according to the following aspects: perioperative outcomes (surgery time, the volume of injected cement, X-ray exposure time, and kyphotic angle reduction); clinical outcomes (visual analogue scale [VAS] for pain relief and Oswestry Disability Index [ODI] for quality of life); and complications (cement leakage and adjacent vertebral fractures).

## 2 Methods

### 2.1 Ethical review

No ethical approval and patient informed consent are required in this article, because all analyses were based on previous published studies.

### 2.2 Search strategy

A search was conducted in Web of Science, PubMed, Embase, and the Chinese Biomedical Database publication databases for all comparative studies published from January 2008 to November 2016 that compared the use of UPKP and BPKP for the treatment of OVCFs. The search terms used included “osteoporotic vertebral compression fractures,” “unilateral balloon kyphoplasty,” “unipedicular balloon kyphoplasty,” “bipedicular balloon kyphoplasty,” “bilateral balloon kyphoplasty,” “bilateral approach,” and “unilateral approach.”

### 2.3 Eligibility criteria

Studies that met the following criteria were included: patients had primary OVCFs; the treatment interventions used were either UPKP or BPKP; the study made comparisons between UPKP and BPKP; outcomes were reported for at least one of the following factors: surgery time, the volume of injected cement, X-ray exposure time, kyphotic angle reduction, cement leakage, adjacent vertebral fracture, VAS, or ODI; and the study was an RCT or cohort study (CS). Studies were excluded if the study was a duplicate publication, a review, or case report; the study-enrolled patients with traumatic fractures, infections, or secondary osteoporosis, such as corticosteroids or endocrine disorders; or the study did not report on the outcomes that were the focus of our meta-analysis.

### 2.4 Data extraction and quality assessment

Two investigators independently collected data from the included studies. Any disagreements were resolved by discussion, or in some cases, a 3rd investigator resolved the conflict. General data from the studies, including first author, year of publication, study design, the number of patients, mean age of the patients (in years), sex ratio (M/F), follow-up (in months), and other outcomes of interest were collected. The assessment of study quality was based on the classic Newcastle Ottawa Scale. Newcastle Ottawa Scale scores ranged from 0 to 9 points, with higher scores indicating better quality.

### 2.5 Statistical analyses

All statistical analyses were conducted using Stata/SE11.0 software (Stata Corporation, College Station, TX). For continuous outcomes, weighted mean difference (WMD) and the 95% confidence interval (CI) were calculated. Risk ratio (RR) and the 95%CI were calculated for dichotomous outcomes. Heterogeneity among studies was assessed using *I*^2^ statistics with judging values <50% (or *P* > .10) indicating acceptable heterogeneity, and values >50% (or *P* < .10) indicating substantial heterogeneity. In the absence of significant heterogeneity, a fixed-effects model was used. Otherwise, a random-effects model was used. For outcome measures, a *P* value <.05 was considered statistically significant. Sensitivity analysis was performed using the “metaninf” command in the Stata/SE 11.0 software. A subgroup analysis was then used to determine the potential source of heterogeneity. In cases in which the data had a large sample size (>10), a funnel plot analysis was applied to determine publication bias.

## 3 Results

### 3.1 Search results and study description

**Figure 1:**
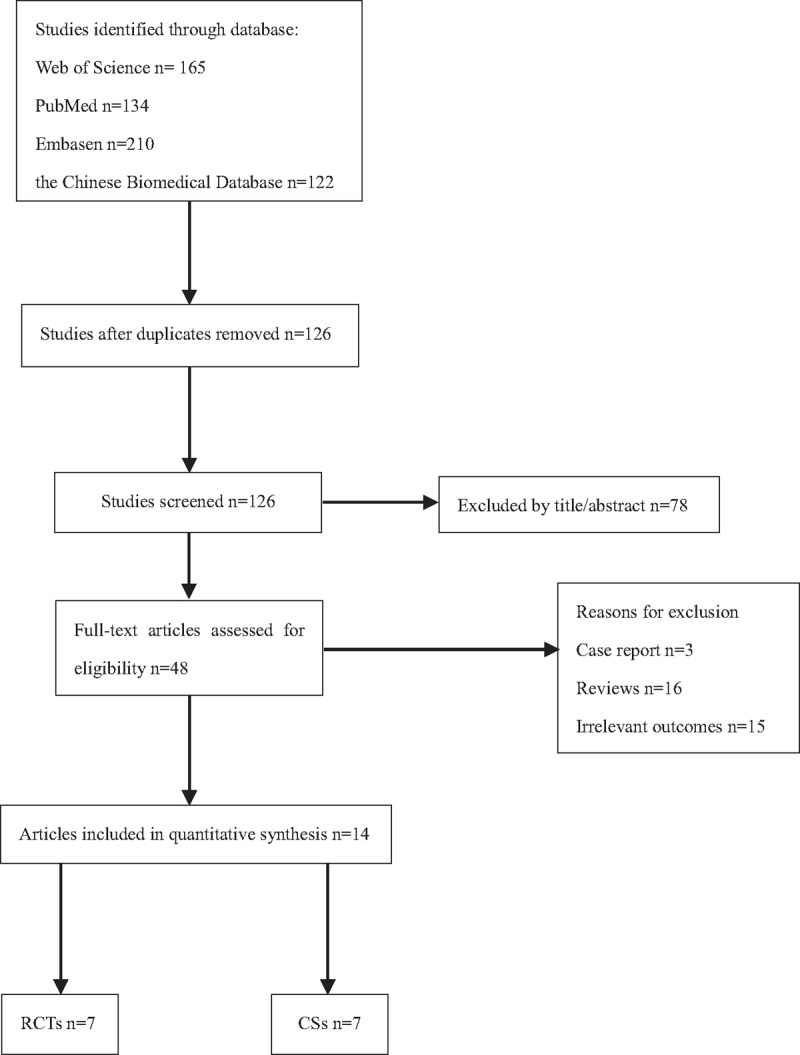
Flow diagram of article selection process.

In total, 631 potential studies in the Web of Science (165), PubMed (134), Embase (210), and the Chinese Biomedical Database (122) publication databases were reviewed. A flow diagram of the article selection process is shown in Fig. 1. Fourteen trials with a total of 1194 patients were retrieved, with the number of patients receiving UPKP and BPKP treatments measuring at 602 and 592, respectively. Descriptions of the basic characteristics of all studies included in this study are listed in Table 1.^[28,18,20,23,26,17,19,27,25,24,22,21]^

**Table 1:**
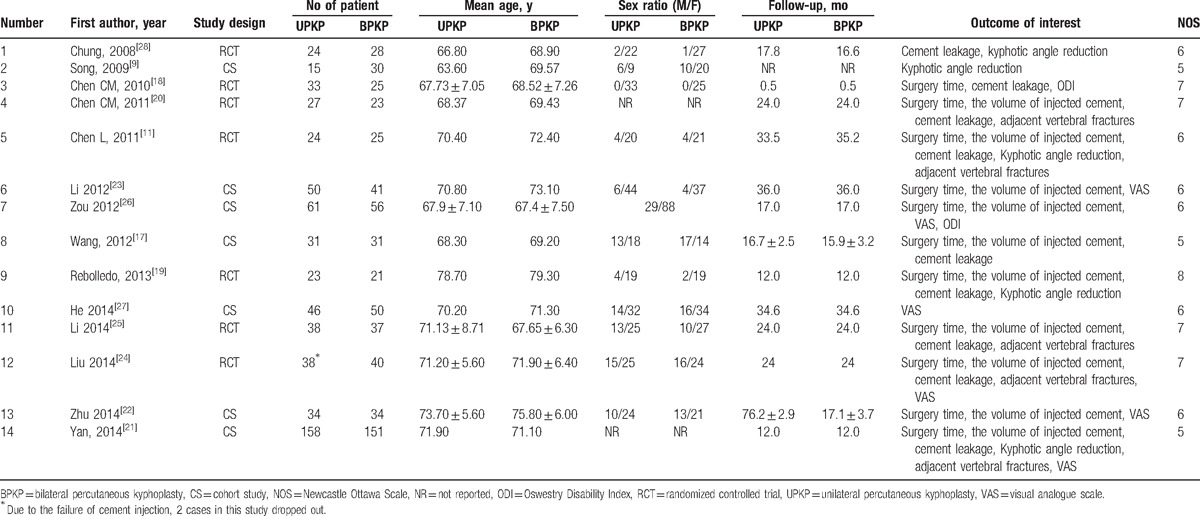
Basic characteristics of included 14 studies.

### 3.2 Outcome measures and data on perioperative aspects

**Figure 2:**
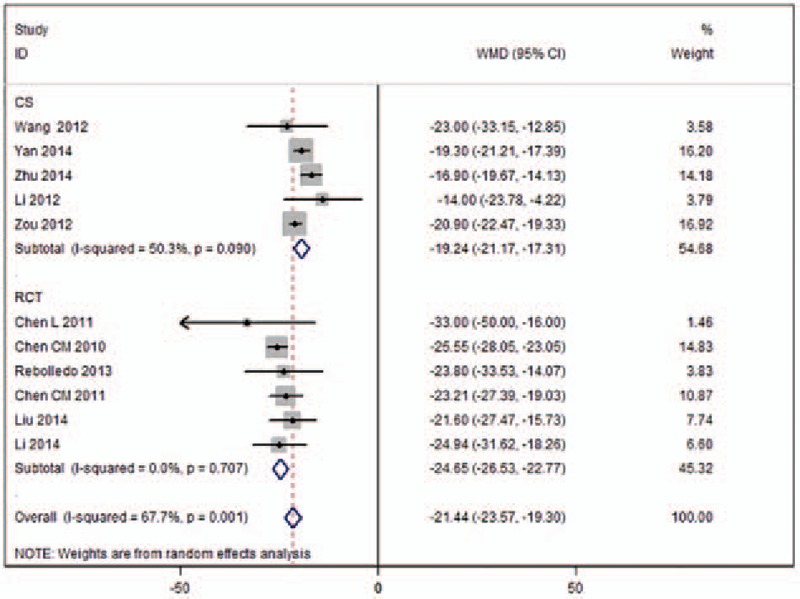
Forest plot of surgery time.

Eleven studies provided data for operation times (Fig. 2).^[11,17–26]^ There was high evidence of heterogeneity (*I*^2^ = 67.7%) across these studies. The pooled data indicated significant differences in surgery time (WMD −21.44, 95%CI [−23.57 to −19.30]; *P* < .001) between UPKP and BPKP. Following subgroup analysis, the results based on the RCTs revealed significant differences in surgery time (WMD −24.65, 95%CI [−26.53 to −22.77]; *P* < .001) between the 2 surgical procedures without heterogeneity (*I*^2^ = 0%) noted between the 2 approaches. The results based on CSs revealed significant differences in surgery time (WMD −19.24, 95% CI [−21.17 to −17.31]; *P* < .001) between the 2 surgical procedures, with significant heterogeneity (*I*^2^ = 50.3%) between the 2 approaches observed (Table 2).

**Table 2:**
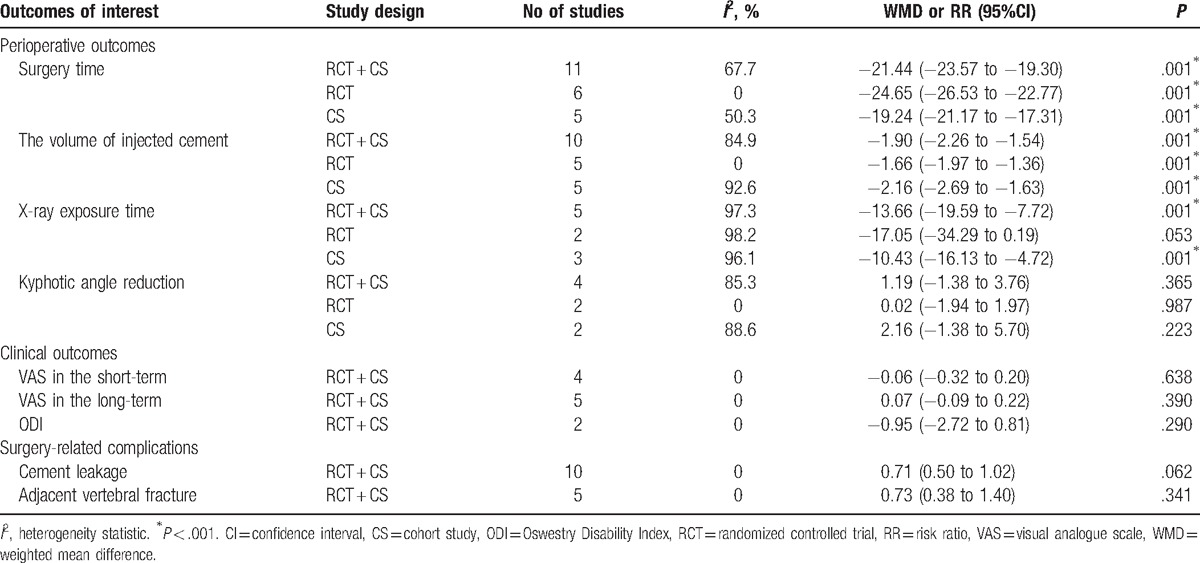
Meta-analysis of all outcomes and subgroup analysis for significant outcomes with the heterogeneity.

**Figure 3:**
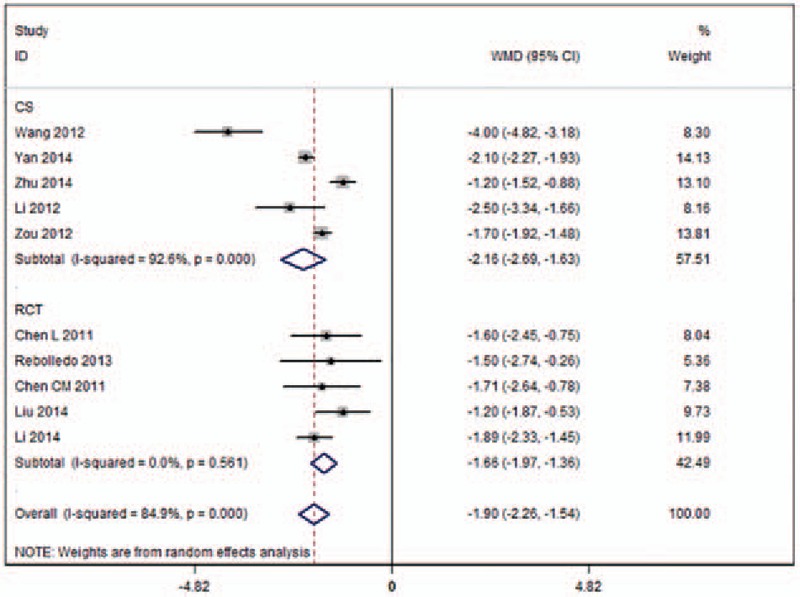
Forest plot of the volume of injected cement.

Ten studies reported on the volume of injected cement (Fig. 3).^[11,17,19–27]^ High heterogeneity (*I*^2^ = 84.9%) across the studies was observed. The pooled results revealed significant differences in the volume of injected cement (WMD −1.90, 95%CI [−2.26 to −1.54]; *P* < .001) between the UPKP and BPKP procedures. After a subgroup analysis, the results based on RCTs indicated significant differences in the volume of injected cement (WMD −1.66, 95%CI [−1.97 to −1.36]; *P* < .001) between the 2 surgical procedures, with was no heterogeneity observed (*I*^2^ = 0%) between the 2 approaches. Results based on CSs revealed significant differences in the volume of injected cement (WMD −2.16, 95%CI [−2.69 to −1.63]; *P* < .001) between the 2 surgical procedures, and substantial heterogeneity (*I*^2^ = 92.6%) between the 2 approaches was observed (Table 2).

**Figure 4:**
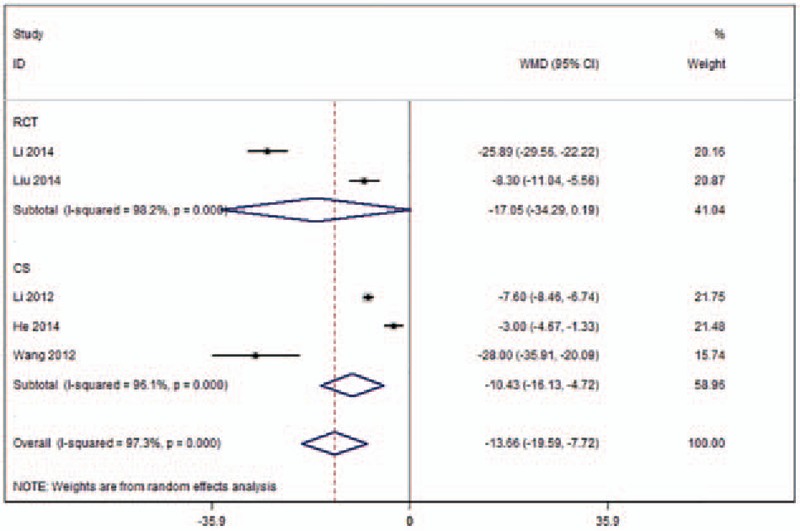
Forest plot of X-ray exposure time.

X-ray exposure time were provided in 5 studies (Fig. 4).^[17,23–25,27]^ Heterogeneity across the studies was 97.3%; therefore, a random-effects model was used. Meta-analysis results demonstrated significant differences in X-ray exposure time (WMD −13.66, 95% CI [−19.59 to −7.72]; *P* < .001) between the 2 surgical procedures. Following subgroup analysis, the identified results based on RCTs indicated that there were significant differences in X-ray exposure time (WMD −17.05, 95%CI [−34.29 to 0.19[; *P* < .001) between the UPKP and BPKP procedures, while the results based on CSs indicated that there were no significant differences in X-ray exposure time (WMD −10.43, 95%CI [−16.13 to −4.72]; *P* = .053) between the 2 surgical procedures. The heterogeneity across the studies after subgroup analysis was more than 96.1% for both the RCTs and CSs (Table 2).

**Figure 5:**
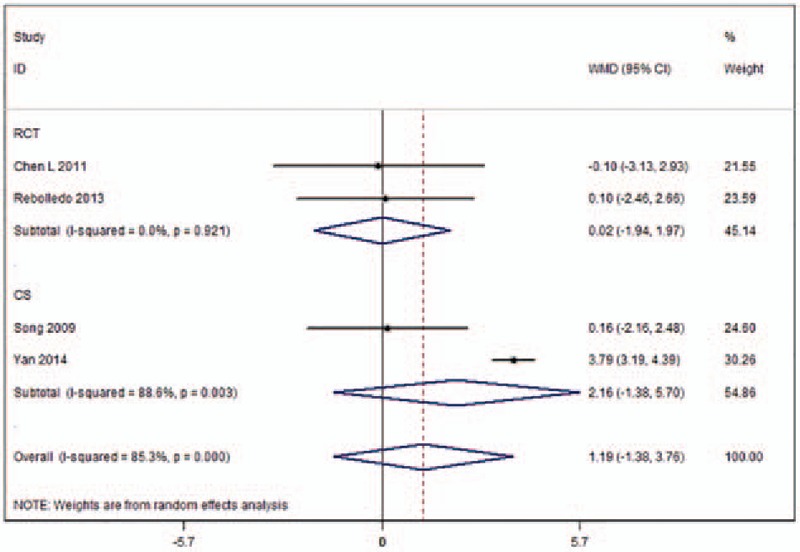
Forest plot of the volume for kyphotic angle reduction.

Kyphotic angle reduction was provided in 4 studies (Fig. 5).^[9,11,19,21]^ Heterogeneity across these studies was 85.3%, so a random-effects model was used. Meta-analysis results demonstrated that there were no differences in kyphotic angle reduction (WMD 1.19, 95%CI [−1.38 to 3.76]; *P* = .365) between the 2 surgical procedures. Following subgroup analysis, the results based on RCTs indicated that there were no significant differences in kyphotic angle reduction (WMD 0.02, 95%CI [−1.94 to 1.97]; *P* = .987) between the UPKP and BPKP procedures without heterogeneity (*I*^2^ = 0%) across the 2 approaches. The results based on the CSs indicated that there were no significant differences in kyphotic angle reduction (WMD 2.16, 95%CI [−1.38 to 5.70]; *P* = .053) between the 2 surgical procedures. The heterogeneity across the studies was 88.6% (Table 2).

### 3.3 Clinical outcomes

**Figure 6:**
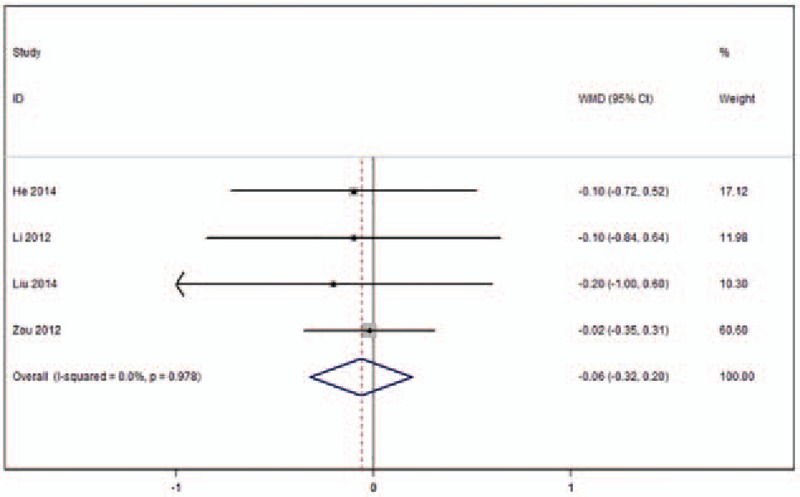
Forest plot of visual analogue scale (VAS) in the short-term (≤1 month follow-up).

Four studies included in this meta-analysis reported VAS in the short-term (ie, a maximum of 1 month of follow-up) (Fig. 6).^[23,24,26,27]^ No heterogeneity across these studies was observed (*I*^2^ = 0%), and no statistically significant differences in VAS after either form of kyphoplasty were found (WMD −0.06, 95%CI [−0.32 to 0.20]; *P* = .638).

**Figure 7:**
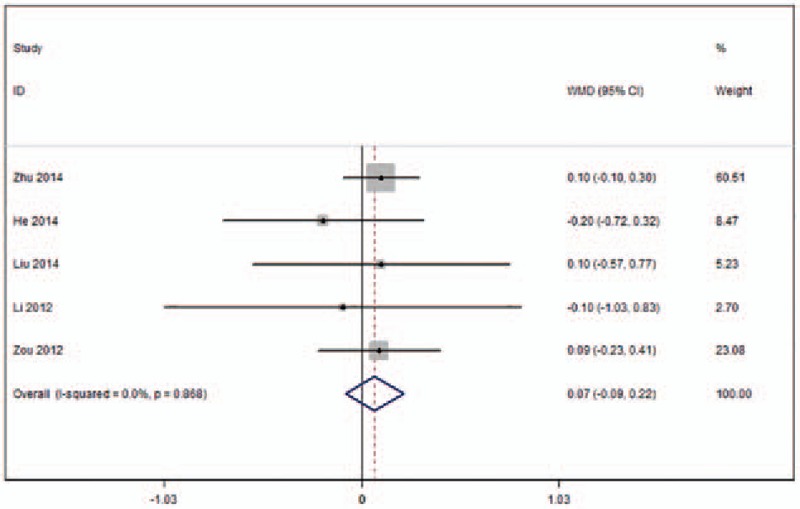
Forest plot of visual analogue scale (VAS) in the long-term (≥1year follow-up).

Five studies were included in this meta-analysis that reported VAS for follow-up periods ≥1 year (Fig. 7).^[22–24,26,27]^ No heterogeneity across these studies was observed (*I*^2^ = 0%) and the pooled results showed no significant differences in VAS for at least 1 year of follow-up (WMD 0.07, 95%CI [−0.09 to 0.22]; *P* = .390) between the 2 surgical procedures.

**Figure 8:**
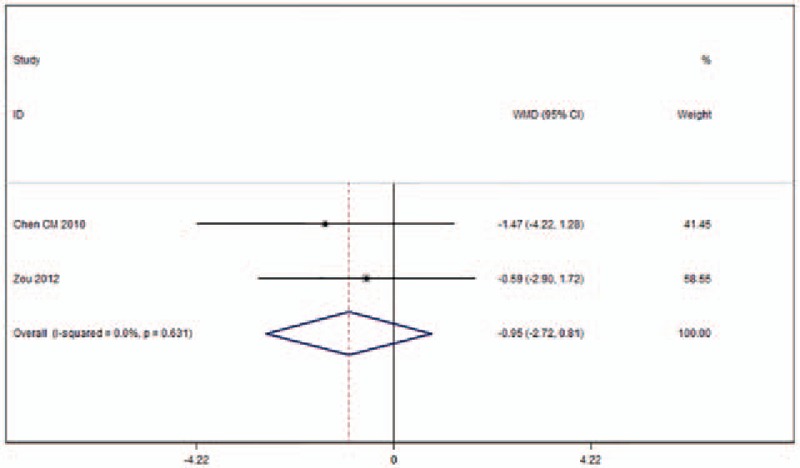
Forest plot of Oswestry Disability Index (ODI).

Regarding ODI, 2 studies met the eligibility criteria for consideration (Fig. 8).^[18,26]^ There was no heterogeneity across these studies(*I*^2^ = 0%). The ODI for UPKP was similar to that for BPKP (WMD −0.95, 95%CI [−2.72 to 0.81]; *P* = .290).

### 3.4 Surgery-related complications

**Figure 9:**
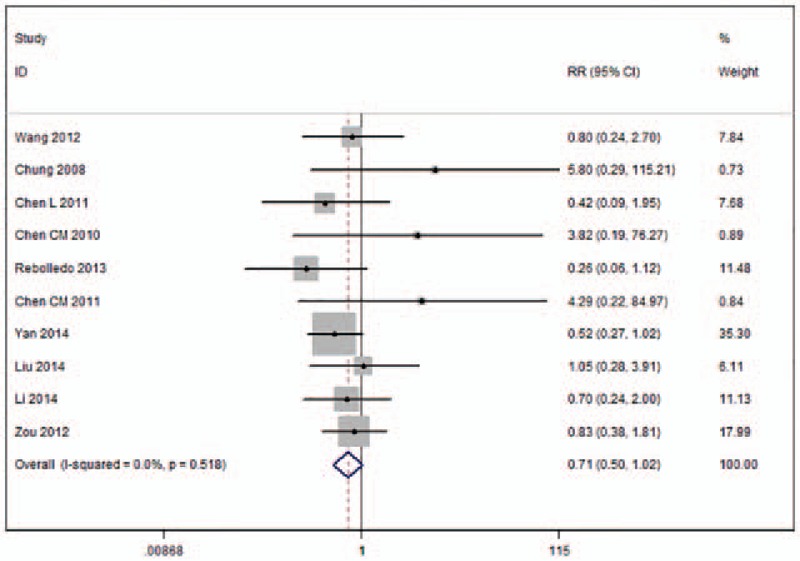
Forest plot for volume of cement leakage.

Ten studies reported occurrences of cement leakage in the 2 procedure groups, with a heterogeneity test showing relatively low statistically significant heterogeneity between the groups (*I*^2^ = 0%) (Fig. 9).^[11,17–21,24–26,28]^ Meta-analysis results indicated that there was no significant difference in occurrence of cement leakage between the 2 groups (RR 0.71, 95%CI [0.50–1.02]; *P* = .062).

**Figure 10:**
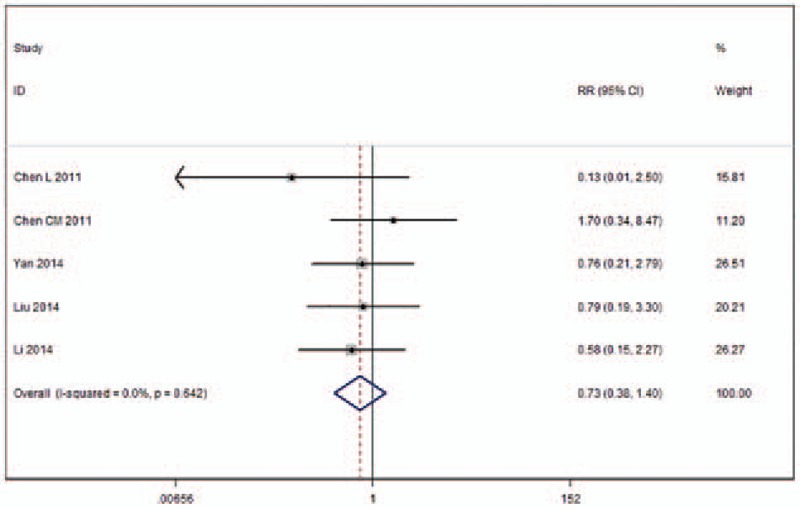
Forest plot for adjacent vertebral fractures.

Five of the included studies reported occurrences of adjacent vertebral fractures (Fig. 10).^[11,20,21,24,25]^ No statistically significant heterogeneity was found between the 2 groups (*I*^2^ = 0%), and a fixed-effects model was applied for meta-analysis. The number of adjacent vertebral fractures among patients receiving UPKP was not higher than among those receiving BPKP (RR 0.73, 95% CI [0.38–1.40]; *P* = .341).

### 3.5 Sensitivity analysis

Sensitivity analysis was performed using the “metaninf” command in Stata/SE 11.0 software. Any single elimination of these studies did not have an influence on the overall outcomes that were assessed.

### 3.6 Publication bias

**Figure 11:**
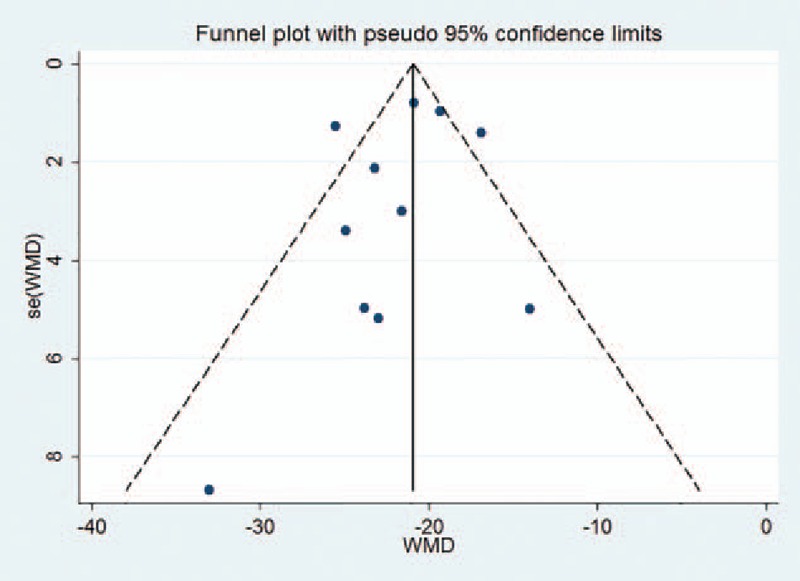
Funnel plot for surgery time.

**Figure 12:**
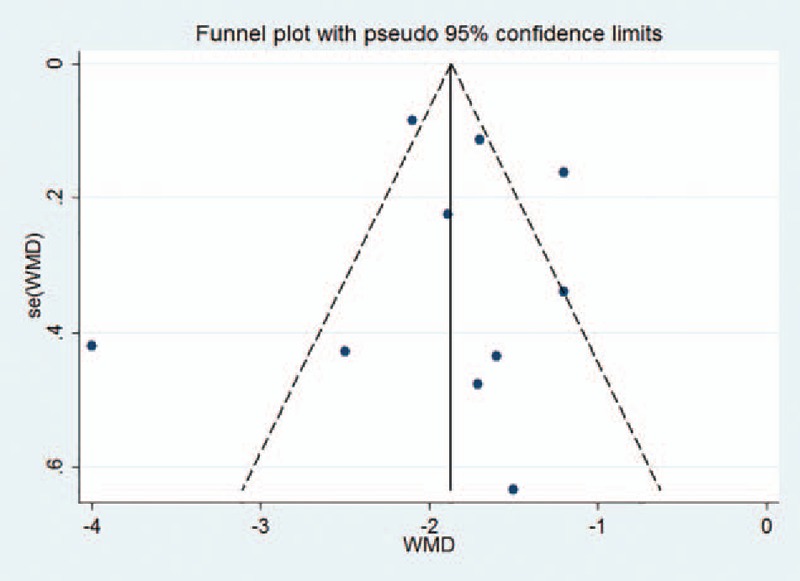
Funnel plot for the volume of injected cement.

Due to the large sample size (≥10) in this meta-analysis, the outcomes that were pooled, such as surgery time and the volume of injected cement, were applicable for funnel plot analysis (Figs. 11 and 12). The funnel plots not only showed asymmetry but also had 2 outliers.

## 4 Discussion

Kyphoplasty is an effective and safe treatment choice for the management of OVCFs in the elderly, and involves realigning the spinal column and restoring the compressed vertebral body height.^[29]^ Specific kyphoplasty treatment options include UPKP and BPKP. Nevertheless, the optimal surgical procedure for OVCFs is still being debated. The objective of our study was to gather as much data as possible to ascertain which procedure (ie, UPKP or BPKP) is superior. Overall, we included 14 studies with 1194 patients in our study. Our meta-analysis found that UPKP was associated with shorter surgery time and lower volumes of injected cement. However, there were no significant differences in VAS, ODI, X-ray exposure time, kyphotic angle reduction, cement leakage, or adjacent vertebral fracture between the 2 surgical procedures.

Compared to UPKP, BPKP theoretically required twice as much cement injection volume, which yielded stronger stability for the vertebral body. Moreover, the stabilization of the vertebral body was probably beneficial to pain relief.^[30,31]^ Therefore, we concluded that the BPKP procedure can produce more satisfactory pain relief and a more significant improvement in quality of life in patients with OVCFs. Both VAS for pain relief and ODI for life quality improvement were used to assess the clinical outcomes of the 2 surgical procedures. However, although we divided the final follow-up times into short-term (≤1-month follow-up) and long-term (≥1-year follow-up) groups, our pooled results did not indicate an advantage of the BPKP procedure over UPKP in terms of either the short-term VAS or the long-term VAS without heterogeneity across the studies. This result was also confirmed in another study by Chen et al^[20]^ that reported that both VAS and ODI for BPKP were similar to that for UPKP.

This study demonstrated that patients undergoing UPKP procedures needed less surgery time, less injected cement, and had lower radiation exposure time compared to those undergoing BPKP. However, there was great heterogeneity between the 2 procedures. To investigate potential sources of heterogeneity, we conducted a sensitivity analysis and found that the single elimination of these studies did not have an influence on the pooled outcomes that were assessed. However, when a subgroup analysis was conducted according to the type of studies that were included, we found that the heterogeneity between the 2 groups disappeared after the removal of the CSs. Based on that analysis, we inferred that the heterogeneity across the 2 procedures in surgery time and the volume of injected cement primarily arose from the presence of the CSs. Nevertheless, the results identified after subgroup analysis demonstrated that the UPKP group had no differences in X-ray exposure time as compared with the BPKP group, which contrasted the result that we obtained before subgroup analysis. Moreover, substantial heterogeneity across all of the studies still existed. Based on our statistical analysis, the potential heterogeneity may not be due to the study design, but could be attributed to the presence of 2 of the RCTs that were included. Thus, we should be very careful in interpreting the meta-analysis results, especially the results that are based on a relatively small number of studies. Finally, we believed that our results were reliable, except for X-ray exposure time. Compared to BPKP procedures, patients undergoing UPKP required less surgery time and less injected cement.

With regard to the type of vertebral body fracture, there were 7 studies reporting anterior wedging, 6 reporting centrally scalloping, and 1 reporting posterior wedging, respectively.^[11,17,22,24–27]^ Because the original studies evaluated did not regard the type of fracture as a confounding factor affecting the efficacy of surgery, therefore, we could not perform the necessary meta-analysis and thus, were not able to determine whether the type of fracture was one of the factors affecting the efficacy of kyphoplasty.

In this study, we did not perform a meta-analysis reporting compressed vertebral body height and its restoration. Due to different definitions of compressed vertebral body height in the included RCTs, we were unable to gather comparable data to evaluate compressed vertebral body height. Khurjekar et al^[32]^ and Muto et al^[33]^ reported that kyphotic angle reduction may be a more important measure than the restoration of compressed vertebral body height in kyphoplasty. The results demonstrated the UPKP group had no more kyphotic angle reduction than as compared with the BPKP group. However, it is worth noting that the heterogeneity across the studies was 85.3%. Although Feng et al^[13]^ also performed a meta-analysis for kyphotic angle reduction with 85% heterogeneity, they did not explore the potential sources of heterogeneity further. Unlike the study by Feng et al, we conducted a subgroup analysis for kyphotic angle reduction and found that the heterogeneity across the studies was 0% after the CSs were removed. Thus, we believed that the heterogeneity was due to CSs. Overall, there were no differences in kyphotic angle reduction and no heterogeneity between the 2 procedures was indicated.

A concern about using cement to treat OVCFs was cement leakage. It was reported that cement leakage through the posterior cortex or the pedicle after removal of the trocar may result in paraparesis.^[34,35]^ The cement injection volume has a positive correlation with cement leakage.^[36]^ In fact, 2 mL of polymethyl methacrylate cement has been shown to have enough power to restore the strength of a compressed vertebral body.^[37]^ Too much cement, however, is a substantial risk factor for cement leakage.^[16]^ In a BPKP procedure, cement is injected through 2 pedicles, which requires twice the volume of cement used in a UPKP procedure. The cemented vertebrae that result can change the biomechanics of the spine and subsequently increase the incidence of new adjacent vertebral fractures.^[38]^ Accordingly, we predicted that patients who had undergone BPKP would have higher incidences of cement leakage and adjacent vertebral fractures as compared to patients who had undergone UPKP. However, our results did not support this hypothesis. We may have ignored possible risk factors, such as injection pressure. Robinson et al^[39]^ compared balloon pressure in fresh fractures and in partially healed compression fractures and found that patients with fresh fractures needed more pressure. Excessive pressure on a partially healed compression fracture was more likely to lead to cement leakage. As such, surgeons should pay close attention to the relationship between the treatment time for the fracture and the pressure of the injected cement.

In terms of surgery time and the volume of injected cement, the funnel plots not only showed asymmetry but also 2 outliers. This publication bias was likely due to the low quality of some of the studies that were included. Additionally, many of the selected studies were conducted in Asia, which may also have resulted in a degree of selection bias.

## 5 Limitations

This study has some limitations. First, low-quality studies that were included might have weakened the strength of our analysis. Second, the type of bone cement was not all described in the selected articles, and we did not analyze this factor at this time. Third, the time from fracture to treatment ranged from less than 1 week to more than 6 months, which may have had an impact on surgical effectiveness.^[18,20,25]^ However, no useful data could be collected and analyzed for the determination of the impact of this factor. Therefore, this study does not report the timing from the presumed onset of the fracture to the treatment on the clinical outcomes. Fourth, the loss of vertebral height assessing the degree of the vertebral body compression was not stated, since the RCTs included in our meta-analysis applied different definitions of compressed vertebral body. It is desirable to have uniform standards for the measurement of vertebral height changes in future RCTs.

## 6 Conclusion

Compared to BPKP, UPKP can achieve similar clinical results for the treatment of OVCFs when assessed in terms of pain relief, improvement of life quality, and presence and significance of surgery-related complications. However, the UPKP procedures evaluated had shorter operation time and a lower volume of injected cement compared to the BPKP procedures. Additional high quality and multicenter RCTs are needed to provide further robust evidence for consideration.
